# Endoscopic Resection Without Subsequent Ablation Therapy for Early Barrett’s Neoplasia: Endoscopic Findings and Long-Term Mortality

**DOI:** 10.1007/s11605-020-04836-8

**Published:** 2020-11-02

**Authors:** S. N. van Munster, E. A. Nieuwenhuis, B. L. A. M. Weusten, L. Alvarez Herrero, A. Bogte, A. Alkhalaf, B. E. Schenk, E. J. Schoon, W. Curvers, A. D. Koch, S. E. M. van de Ven, P. J. F. de Jonge, T. Tang, W. B. Nagengast, F. T. M. Peters, J. Westerhof, M. H. M. G. Houben, Jacques J. G. H. M. Bergman, R. E. Pouw, on behalf of the Dutch Barrett Expert Centers

**Affiliations:** 1 Department of Gastroenterology and Hepatology, Amsterdam Gastroenterology Endocrinology and Metabolism, Amsterdam University Medical Centers, location AMC, Meibergdreef 9, 1105 AZ Amsterdam, The Netherlands; 2 Department of Gastroenterology and Hepatology, Sint Antonius Hospital, Nieuwegein, The Netherlands; 3 Department of Gastroenterology and Hepatology, University Medical Center Utrecht, Utrecht University, Utrecht, The Netherlands; 4 Department of Gastroenterology and Hepatology, Isala Clinics, Zwolle, The Netherlands; 5 Department of Gastroenterology and Hepatology, Catharina Hospital, Eindhoven, The Netherlands; 6 Dept. of Gastroenterology and Hepatology, Erasmus Medical Center, Rotterdam, The Netherlands; 7 Departmant of Gastroenterology and Hepatology, IJsselland Hospital, Capelle aan den Ijssel, The Netherlands; 8 Department of Gastroenterology and Hepatology, University Medical Center Groningen, Groningen University, Groningen, The Netherlands; 9 Department of Gastroenterology and Hepatology, Haga Teaching Hospital, Den Haag, The Netherlands

**Keywords:** Barrett’s esophagus, Esophageal adenocarcinoma, Endoscopic therapy, Endoscopic mucosal resection

## Abstract

**Introduction:**

After endoscopic resection (ER) of neoplasia in Barrett’s esophagus (BE), it is recommended to ablate the remaining BE to minimize the risk for metachronous disease. However, we report long-term outcomes for a nationwide cohort of all patients who did not undergo ablation of the remaining BE after ER for early BE neoplasia, due to clinical reasons or performance status.

**Methods:**

Endoscopic therapy for BE neoplasia in the Netherlands is centralized in 8 expert centers with specifically trained endoscopists and pathologists. Uniformity is ensured by a joint protocol and regular group meetings. We report all patients who underwent ER for a neoplastic lesion between 2008 and 2018, without further ablation therapy. Outcomes include progression during endoscopic FU and all-cause mortality.

**Results:**

Ninety-four patients were included with mean age 74 (± 10) years. ER was performed for low-grade dysplasia (LGD) (10%), high-grade dysplasia (HGD) (25%), or low-risk esophageal adenocarcinoma (EAC) (65%). No additional ablation was performed for several reasons; in 73 patients (78%), the main argument was expected limited life expectancy. Median C2M5 BE persisted after ER, and during median 21 months (IQR 11–51) with 4 endoscopies per patient, no patient progressed to advanced cancer. Seventeen patients (18%) developed HGD/EAC: all were curatively treated endoscopically. In total, 29/73 patients (40%) with expected limited life expectancy died due to unrelated causes during FU, none of EAC.

**Conclusion:**

In selected patients, ER monotherapy with endoscopic surveillance of the residual BE is a valid alternative to eradication therapy with ablation.

## Introduction

Barrett’s esophagus (BE) is the most important risk factor for esophageal adenocarcinoma (EAC), which has a poor prognosis. Identifying EAC at an early stage allows for endoscopic treatment with an excellent prognosis. The first step in endoscopic treatment for BE-related neoplasia is removal of all visible lesions with endoscopic resection (ER) techniques, which serves both diagnostic and therapeutic purposes. It has been reported that the remaining flat BE that persists after ER of a neoplastic lesion has a risk of developing metachronous HGD/EAC between 15 and 30% in 3–5 years 1–3. Based on these data, most international guidelines advise additional ablation therapy after ER to eradicate the entire BE segment 4–7. Given the large amount of high-quality data supporting radiofrequency ablation (RFA), this is recommended as first-choice ablation technique 4, 8–10.

Although RFA therapy is highly effective for eradication of flat BE, the choice to continue with ablation requires balanced decision-making, taking into account patient’s age, comorbidity, and life expectancy 6. The aforementioned FU studies have also shown that metachronous lesions were always detected at early stages that allowed curative endoscopic treatment 1–3. Moreover, the majority of patients will never develop metachronous neoplasia. Performing RFA for all post-ER patients may thus be associated with overtreatment.

Although severe complications due to RFA treatment are very rare, complications do occur, most commonly esophageal strictures in up to 10–14% 11. Furthermore, RFA treatment to eradicate all residual BE requires on average 3 additional therapeutic endoscopies. Patients may experience post-procedural pain, discomfort, or dysphagia. Therefore, after ER for early neoplasia, endoscopic surveillance (“ER monotherapy”) may be an acceptable alternative to RFA, especially in patients with older age and/or severe comorbidities.

In the Netherlands, endoscopic treatment for BE is centralized in 8 Barrett Expert Centers (BECs), with a uniform treatment and follow-up protocol. Since the introduction of RFA in 2008, these centers adhered to the ER monotherapy strategy in selected patients. In the current study, we report the long-term outcomes of “ER monotherapy” as an alternative to additional ablation therapy in patients with limited life expectancy.

## Methods

This study was based on the Barrett Expert Center registry (BEC registry) (Netherlands Trial Register, NL7039), which has been described in detail earlier 12. In short, this registry captures outcomes for all patients with Barrett’s neoplasia in the Netherlands who underwent endoscopic treatment since 2008. Care for BE neoplasia in the Netherlands is centralized in 8 Barrett Expert Centers (BECs), with the implication that every patient in the Netherlands is treated in one of these expert centers. This centralized organization of care was established in 2007. At that moment, a joint training program was launched for endoscopists and pathologists, one of both from each center. All BE care in the Netherlands since then has been provided by the specifically trained endoscopists and pathologists. The BECs adhered to a common treatment and follow-up protocol, and several meetings a year were held to further guarantee homogeneity. Apart from this close collaboration for clinical care, a solid joint research infrastructure was founded and resulted in multiple publications in the field of pathology 13–16, imaging 17–19, and treatment 8, 9, 20–26 of early BE neoplasia.

The centers have a minimum annual case load of 10 new patients with neoplasia per year, and all new cases are registered in a database.

### Treatment Protocol

Patients were referred to a BEC for careful work-up and staging after being diagnosed with low-grade dysplasia (LGD), HGD, or EAC. During an upper gastrointestinal endoscopy (UGE), the esophagus was carefully inspected with documentation of the Prague C&M criteria and presence of visible lesions or other abnormalities such as esophagitis or esophageal stenosis.

If a visible abnormality was detected, endoscopic resection (ER) was performed for histologic staging using the ER-cap technique, multiband mucosectomy (MBM), or endoscopic submucosal dissection (ESD) per physician’s discretion. Four-quadrant random biopsies were obtained from the (residual) flat BE segment according to the Seattle protocol 27.

If the ER specimen showed LGD, HGD, or low-risk (LR) EAC (defined as ≤sm1 invasion with good to moderate differentiation, without lymphovascular invasion and with radical vertical resection margin), a balanced decision was made between further endoscopic treatment or surveillance. In the vast majority of patients, additional ablation therapy was offered to achieve a complete eradication of the entire Barrett’s segment. However, in patients with limited life expectancy, for example, due to older age and/or severe comorbidity, surveillance of the remaining BE was preferred with endoscopic intervention in case of recurrent neoplasia and/or visible lesions.

All patients were prescribed bi-daily high-dose proton-pump inhibitors.

### Follow-Up Protocol

FU for persisting non-dysplastic BE (NDBE)/LGD after ER consisted of yearly surveillance endoscopies in year 1 to 5, and then once per 2–3 years. FU was performed every 3–6 months for persisting HGD. The decision to stop further surveillance was made per physician’s discretion in agreement with the patient.

### Study Population

For the current study, we included all patients from the BEC registry who underwent ER monotherapy for LGD, HGD, or LR-EAC with residual flat BE before January 1, 2018.

### Study Endpoints

The first primary endpoint was progression to HGD/EAC in the remaining BE. For patients with remaining NDBE or LGD, detection of HGD/EAC was considered to be progression. For patients with persisting flat HGD, new EAC was progression as was a new visible lesion containing HGD. All patients were included for this analysis. This endpoint was stratified for residual grade of dysplasia.

The second primary endpoint was all-cause mortality. This endpoint reflects whether the decision to prefer surveillance over ablation was justified for patients with expected limited life expectancy. Therefore, only the patients in whom the decision for ER monotherapy was based on age and/or comorbidity were included for this analysis.

Secondary endpoints included symptomatic EAC and/or EAC-related death and predictors for progression. All patients were included for these analyses. We also assessed progression risk to HGD/EAC in the remaining BE among only patients who had at least 18 months of endoscopic FU.

### Data Collection and Data Management

Endoscopy and pathology data were collected in standardized form in all BECs, by medical students in the final year of their degree. Additionally, all patients with endpoints and an additional 50% of the remaining patients were double-checked by dedicated research fellows (all MDs). All fields were examined for missing data, unlogical values, or outliers, with data being completed or corrected where possible.

The BEC registry was merged with the non-public microdata from Statistics Netherlands for date and cause of death.

### Statistics

Continuous variables were presented as mean with standard deviation (SD) or median with interquartile range (IQR) for normally distributed or skewed data, respectively. Categorical variables were presented as numbers with percentages, and 95% confidence intervals (CI) were obtained using internal bootstrapping.

Progression risks were plotted using the cumulative incidence curve (CII), taking competing risks of unrelated death into account. Annual progression rates were calculated as the number of progressors divided by the total follow-up duration in years. Predictors for progression were assessed using Cox regression and Fine and Gray competing risk analysis, the latter considered unrelated death as competing risk.

Statistical analysis was performed using Rstudio for Windows (version 3.6.1) and packages: survival, survminer, cmprsk, ggplot2, and Hmisc.

### Ethics

The Institutional Review Board of the Amsterdam University Medical Centers declared that the registry was not subject to the Medical Research Involving Human Subjects Act (“wet op medisch-wetenschappelijk onderzoek met mensen” in Dutch) and waived the need for formal ethical review and patient-informed consent. Patients were approached through an opt-out card with the possibility to object against participation in the registry.

## Results

### Patient Description

**Fig. 1:**
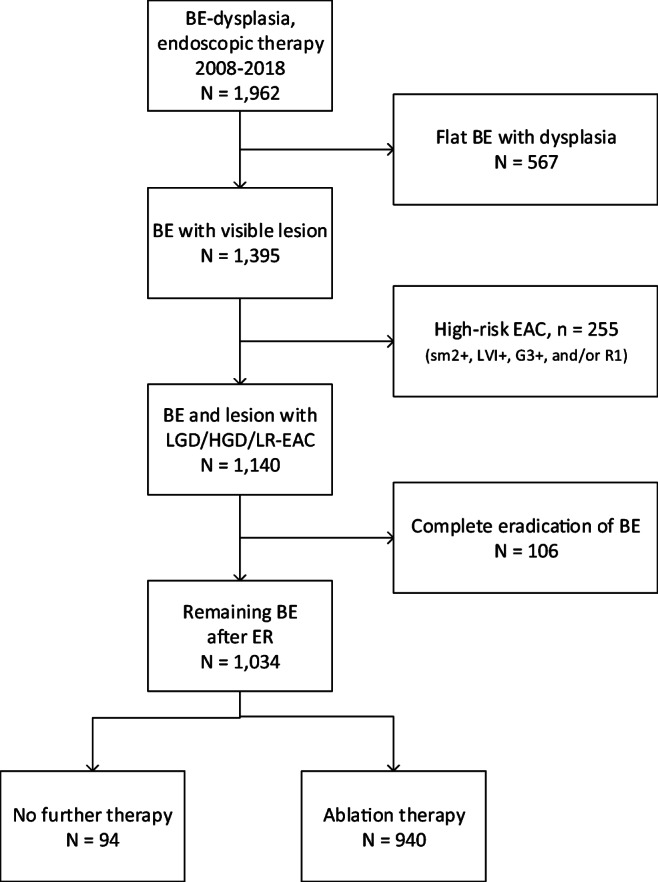
Flowchart. Patient flow in the Barrett Expert Center Registry. All patients with remaining flat BE after ER for which no ablation was performed were included in the current study (*n* = 94)

Between 2008 and 2018, a total of 1962 patients with early BE neoplasia were referred to a BEC. A visible abnormality was detected in 1395 patients (71%) and removed with ER (Fig. 1). After ER for LGD, HGD, or LR-EAC (*n* = 1140), a flat BE segment remained in 1034 patients. The vast majority of these patients (91%) underwent additional ablation aimed at eradication of the entire BE segment. Ninety-four patients (9%) had ER monotherapy for LGD (*n* = 9), HGD (*n* = 23), T1a EAC (*n* = 47), or T1bsm1 EAC (*n* = 15), with remaining BE, and were included for this study.

Patients had a mean age of 74 (± 10) years and ASA classification II (67%) or III/IV (23/2%), with ER performed for LGD (10%), HGD (25%), or LR-EAC (66%) (Table 1).

**Table 1:** Demographics

	All *N* = 1034	ER monotherapy *N* = 94	ER + ablation *N* = 940
Male gender, *n* (%)	803 (80)	64 (86)	766 (82)
Age, years (mean (± SD))	66 (10)	74 (10)	65 (9)
BMI, kg/m^2^ (mean (± SD))	28 (5)	27 (10)	27 (5)
ASA classification, *n* (%)			
I		7 (7)	
II		63 (67)	
III		22 (23)	
IV		2 (2)	
Smoking, *n* (%)			
Current	169 (23)	12 (18)	157 (23)
Former	338 (46)	37 (56)	301 (45)
Never	232 (31)	17 (26)	215 (32)
Esophagitis, *n* (%)	36 (4)	2 (2)	34 (4)
BE segment, cm (median (IQR))	C2M5 (1–5; 3–7)	C4M6 (1–7; 3–9)	C2M5 (1–5; 3–7)
Lesion, Paris-type, *n* (%)			
0-Ip/s	107 (13)	17 (24)	90 (12)
0-IIa	563 (67)	35 (50)	528 (69)
0-IIb	131 (16)	15 (21)	116 (15)
0-IIc	35 (4)	3 (4)	32 (4)
Lesion, size, mm (mean (± SD))	25 (15)	27 (21)	25 (15)
Lesion, circ extent, % (mean (± SD))	39 (21)	39 (21)	39 (21)
ER type, *n* (%)			
EMR	983 (95)	85 (90)	898 (96)
ESD	49 (5%)	7 (7)	42 (5)
Both	2 (0.2%)	2 (2)	0
Specimen ER, *N* (median (IQR))	2 (1–3)	2 (1–3)	2 (1–3)
< 50% squamous regression of ER-site, *n* (%)	53 (5)	12 (13)	41 (5)
Worst ER pathology, *n* (%)			
LGD	69 (7)	9 (10)	60 (6)
HGD	263 (25)	23 (25)	240 (26)
EAC	702 (68)	62 (66)	640 (68)

*Missing values existed for the following variables (*n* = missing in total cohort/missing in ER monotherapy cohort): BMI (*n* = 164/25), ASA (*n* = 700/0), smoking (*n* = 295/28), Paris classification (*n* = 198/24), and regeneration of ER site (*n* = 56/0)

*ASA* American Society of Anesthesiologists, *BE* Barrett esophagus, *circ* circumferential, *EAC(-m/sm)* esophageal adenocarcinoma (mucosal/submucosal), *EMR* endoscopic mucosal resection, *ER* endoscopic resection, *ESD* endoscopic submucosal dissection, *HGD* high-grade dysplasia, *IQR* interquartile range, *LGD* low-grade dysplasia, *SD* standard deviation

### Decision-Making After ER

After ER for all visible abnormalities, a flat BE segment of median C2M5 (0–5; 3–8) remained with NDBE (*n* = 48, 51%), LGD (*n* = 29, 32%), or HGD (*n* = 6, 6%). In 11 patients (12%), no biopsies were obtained since this was considered not to change clinical decision-making.

**Fig. 2:**
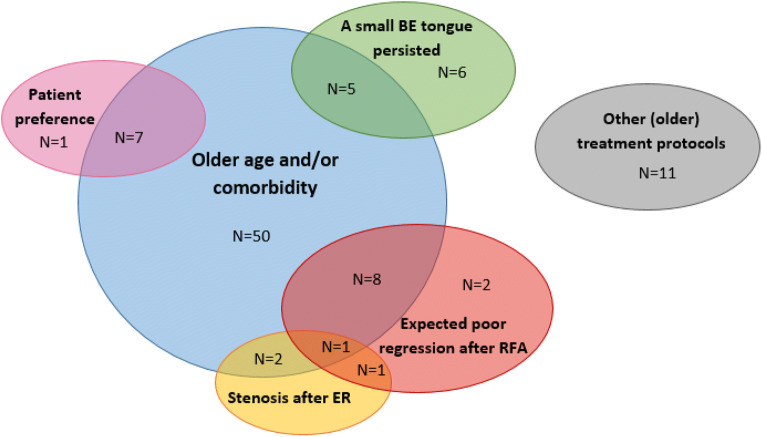
Reasons not to continue with ablation therapy after ER. Several reasons were reported why RFA was not initiated; age and/or comorbidity constituted the most common reasons

In 73 patients (78%), additional ablation was not started due to age and/or comorbidity. Concomitant reasons in this group were as follows: expected poor regression after RFA due to regeneration with BE after ER (*n* = 8, 11%); patient preference (*n* = 7, 10%); persistence of a small BE tongue only (*n* = 5, 7%); and/or complications after ER (*n* = 3, 4%) (Fig. 2).

In the remaining 21 patients (22%) in whom age and comorbidity played no role, reasons not to continue with ablation therapy were as follows: other treatment protocols (e.g., in the pre-RFA era) (*n* = 11, 52%); persistence of a small BE tongue only (*n* = 6, 29%); expected poor regression after RFA due to BE regeneration after ER (*n* = 3, 14%); complications after ER (*n* = 1, 5%); and/or patient preference (*n* = 1, 5%).

### Progression During Follow-Up

During a median endoscopic FU of 21 months (11–51) with a median of 4 endoscopies 3–5 per patient, no patient progressed to advanced cancer. Overall, 17 patients (18%, annual progression risk 8.0% [95% CI 5.1–12.5]) progressed to HGD (*n* = 10) or LR-EAC (*n* = 7) (Table 2). The median time to progression was 26 months (23–47), and the first progression was detected 18 months after ER. All patients who progressed had undergone at least 2 FU endoscopies without abnormalities after ER.

**Table 2:** Progression rates per histologic stage in the remaining BE after ER

Histology in residual BE after ER	Total patients (*N*)	Median FU duration (months)	Median *N* endoscopies	Worst histology during FU					Annual progression rate [95% CI]
				No FU performed	NDBE	LGD	HGD	LR-EAC	
No biopsies	11	17 (8–65)	3 (1–6)	5	5	1	0	0	–
NDBE	48	20 (10–48)	3 (1–5)	0	37	3	3	5	6.4% [3.3–12.1]
LGD	29	22 (14–60)	3 (1–5)	1	13	9 (4*)	5	1	6.7% [3.1–13.9]
HGD	6	41 (14–62)	7 (2–10)	0	0	0	5 (2**)	1	14.5% [5.0–34.6]
Total	94	21 (11–51)	3 (1–5)	6	54	13	13	7	8.0% [5.1–12.5]

Risk for progression to HGD/EAC during endoscopic FU, stratified for histology of the flat BE that remained after ER

*4 with persisting LGD were treated with RFA

**2 with HGD developed a lesion during FU and were treated with ER and counted as progression

Sixteen out of seventeen progressors were successfully treated endoscopically, either with ER for a visible lesion containing LR-EAC (*n* = 7) or HGD (*n* = 6) or with ablation therapy for flat HGD (*n* = 3). A single patient who progressed from LGD to HGD had no further treatment, and the patient died shortly after due to an unrelated cause. Six progressors had developed a worse histological grade during FU, than the initial histology after baseline ER. This included baseline LGD to m-EAC in FU (*n* = 1), baseline HGD with m-EAC in FU (*n* = 4), and baseline m-EAC with sm-EAC during FU (*n* = 1).

The annual risk for progression was 6.4% for residual NDBE and 6.7% for LGD, as compared to 14.5% for residual HGD (Table 2).

In total, 55 patients had an endoscopic FU > 18 months with an annual risk for progression of 8.6% per person year [95% CI 5.4–13.3]. The median FU in this subgroup of patients was 31 months after ER (IQR 17–53).

In the majority (27/39; 69%) of the patients with FU < 18 months, endoscopic FU was discontinued at median 3 months (IQR 0–9) after ER, due to limited life expectancy. Of these 27 patients, 15 had unrelated death median 18 months after ER, whereas the remaining 12 were alive and asymptomatic at median 55 months after ER. The remaining 12/39 patients with short FU were recently treated with ER and were still under endoscopic surveillance (median 12 months).

### All-Cause Mortality

Our second aim was to asses all-cause mortality during long-term follow-up in the subgroup of patients with older age and/or comorbidity, to verify whether ER monotherapy was justified in this group of patients.

**Fig. 3:**
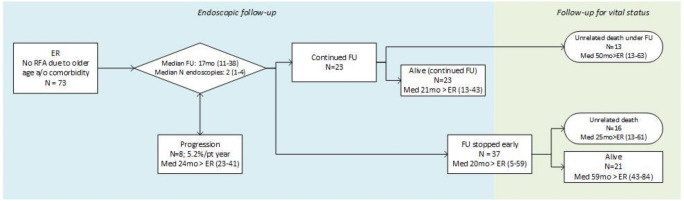
Long-term outcomes for 73 patients with no ablation due to older age and/or comorbidity. The outcomes during endoscopic follow-up, and long-term follow-up for vital status, among the 73 patients where RFA was not initiated due to older age and/or comorbidity

As reported, in 73 patients, age and/or comorbidity played an important role in the decision not to continue with ablation therapy after ER. In 37 patients, endoscopic surveillance was stopped early at median 20 months (5–59) after ER (Fig. 3). Unrelated death occurred in 16 of these patients median 10 months after FU was stopped. The remaining 21 patients were still alive and asymptomatic median 24 months after FU was stopped.

In the remaining 36 patients, endoscopic FU was not stopped early. A total of 13 patients died from unrelated causes while being under surveillance, median 50 months after ER. The remaining 23 patients were still under surveillance at the moment of data collection, median 21 months after ER.

**Fig. 4:**
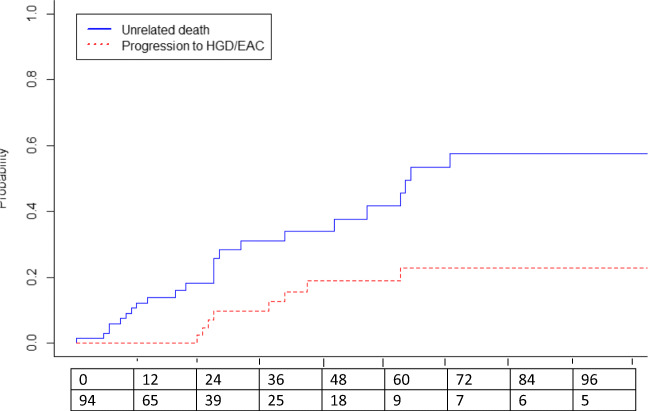
Risk for progression to HGD/EAC and unrelated death after ER monotherapy. Cumulative incidence curves for progression to HGD/EAC and unrelated death during long-term FU after ER monotherapy, among the 73 patients in which RFA was not initiated due to older age and/or comorbidity

Overall, 29 of 73 patients (40%) died due to unrelated causes median 28 months after ER at a median age of 80 (72–85) years. The remaining 44 of 73 patients were still alive at the moment of data collection median 42 months after ER. Figure 4 shows the cumulative incidence curves for progression and unrelated death. Neoplasms other than EAC (*n* = 11, 38%) and cardiovascular disease (*n* = 11, 38%) contributed the most common causes of death.

### EAC-Related Death

None of the 94 patients progressed to disease stages that exceeded boundaries for curative endoscopic treatment, developed symptomatic EAC, or died from EAC.

### Predictors

In univariable analysis, length of the residual BE was significantly associated with risk for progression during FU (Table 3). For patients with a remaining circumferential BE of 0–1 cm, 2–5, or > 5 cm, the annual progression risks were 1.8%, 7.0%, and 15.9%, respectively. The risk increased with 11% for every centimeter increase in BE length. The hazard ratio for persisting HGD versus LGD or NDBE was considerable, but did not reach the level of statistical significance. Estimated hazard ratios for Fine and Gray and Cox analysis were comparable.

**Table 3:** Univariable analysis for progression

		No progression *N* = 77	Progression *N* = 17	FG HR [95% CI]	Cox HR [95% CI]
Demographics	Age, years (mean (± SD))	75 (9)	71 (11)	1.01 [0.94; 1.04]	1.01 [0.95; 1.04]
	Male gender, *n* (%)	52 (68)	12 (71)	0.82 [0.43;3.47]	0.91 [0.32; 2.60]
Baseline BE	Worst ER histology, *n* (%)			2.53 [0.29; 22.3]	1.90 [0.25; 14.67]
	LGD	8 (10)	1 (6)		
	HGD/EAC	69 (90)	16 (94)		
	N ER specimen	2 (1–3)	2 (1–3)	0.94 [0.67; 1.30]	0.99 [0.72; 1.36]
Residual BE	Circumferential extent, cm (median (IQR))	2 (0–5)	5 (2–6)	1.11 [1; 1.23]	1.22 [1.08; 1.40]
	Maximum extent, cm (median (IQR))	5 (2–8)	6 (5–8)	1.09 [0.98; 1.21]	1.13 [0.98; 1.30]
	Worst histology, n (%)			2.17 [0.77; 6.18]	2.47 [0.68; 8.99]
	NDBE/LGD	63 (82)	14 (82)		
	HGD	3 (4)	3 (18)		
	No histology	11 (14)	–		

Univariable analysis for demographic and treatment characteristics for prediction or progression to HGD or EAC during endoscopic FU, assessed with Cox analysis and Fine and Gray estimation

*EAC* esophageal adenocarcinoma, *ER* endoscopic resection, *ER* endoscopic resection, *FG* Fine and Gray, *HGD* high-grade dysplasia, *IQR* interquartile range, *LGD* low-grade dysplasia, *NDBE* non-dysplastic Barrett esophagus, *SD* standard deviation

## Discussion

We report endoscopic and long-term all-cause mortality outcomes for all patients with ER monotherapy in the Netherlands between 2008 and 2018, to assess whether this is a justified treatment strategy in selected patients with early BE neoplasia, for example, in case of older age and/or significant comorbidity. The risk for progression to HGD or EAC was 8% per year. In all cases, progression was detected at early stages and curatively treated endoscopically. No patient developed advanced EAC, and no patient died due to EAC, even though endoscopic surveillance was stopped early in half of the patients. Overall, 40% of patients died due to EAC unrelated causes at median 28 months after ER. These data suggest that ER monotherapy with endoscopic surveillance of the residual BE is a valid alternative to prophylactic ablation therapy in selected patients.

Data from the current study comport well with older studies from the pre-ablation era, reporting progression rates in remaining flat BE after ER varying from 15 during 5 years to 30% in 3 years 1–3. These data have generally been used to justify initiating ablation therapy after ER, but one could also look at it from a different point of view. During every year of FU after ER, only 8% of patients develop progression, and this was always curatively treated with a single ER.

RFA is effective and can achieve complete eradication of all BE (CE-BE) in 90–95% of patients. However, RFA is associated with multiple hospital visits and a risk of complications. Patients with baseline ER have the highest risk for post-RFA stenosis 28. Apart from RFA-related complications, a recent study showed that the risk for cardiovascular complications due to sedation increases with age 29. Unfortunately, we could not evaluate these endpoints in the current study given its retrospective nature with a risk for underreporting of these complications.

The decision to initiate prophylactic ablation therapy of residual BE after ER of neoplasia should be based on the answers to the following three questions:

1. What is the risk for this patient to develop recurrent neoplasia, with or without ablation therapy?

A substantial proportion of patients will never develop neoplasia in the remaining BE after ER. If the remaining BE after ER contains NDBE or LGD, the annual progression risk was only 6.4–6.7%. The median BE length in the current study was C2M5, and for shorter BE lengths, this annual risk will be even lower 1. Apart from the annual risk, we should also consider the cumulative risk for progression. Assume we continue surveillance until the age of 80 years, then the cumulative risk for a 50-year-old patient will be much higher as compared to a 78-year-old patient. Furthermore, if RFA treatment is initiated, it is important to realize that the risk for future neoplasia is lowered, but not reduced to zero. RFA generally fails to achieve CE-BE in 5–10% of patients, and the annual risk for recurrent neoplasia after CE-BE is 0.8% 12.

2. If this patient develops progression, what is the risk of dying from EAC?

Second, the clinically relevant endpoint that should be prevented is progression to advanced, symptomatic EAC and/or EAC-related death. Most endoscopic studies define recurrent HGD or worse as an endpoint, or even recurrent LGD. Although this might be a logic endpoint for some studies, this is not the relevant endpoint that matters to a patient.

**Fig. 5:**
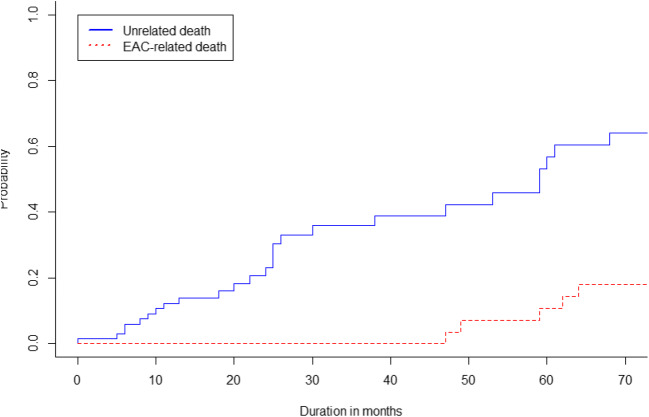
Hypothetical risks for EAC-related death and unrelated death in the situation where we would not have treated progression. Imaginary cumulative incidence curves for EAC-related death and unrelated death. The incidence curve for EAC-related was derived from the assumption that progression that was left untreated would cause EAC-related death 2 years later

Proper data describing the natural history of HGD or early EAC is lacking, but older studies report incidence rates of HGD to advanced EAC of 6.6% per patient year with 2- to 5-year duration between detection of HGD and development of advanced EAC 30, 31. Based on a worst-case scenario in which all patients would have died due to advanced EAC 2 years after progression was detected but not treated, we adapted the factual curve for progression from our study by horizontally shifting it two years to the right (Fig. 5). This new curve now represents the imaginary incidence for EAC-related death in the study population. This plot is based on numerous assumptions and should not be adopted for truth, but merely provides insight in the differences of occurrence and timing for varying endpoints used.

3. How does the risk for EAC-related death relate to the risk of death due to other causes?

Finally, the benefits of eradication of all BE over removal of neoplasia should be balanced against the assumed life expectancy of a patient. Differences in life expectancy would not only change the cumulative risk for recurrence as described above but would also change the curve for unrelated death in Fig 5. For young and fit patients, this curve would shift downwards, whereas it would be steeper along with increasing age and/or comorbidity. The actual decision to eradicate the remaining BE after ER should be based on a balance between the risk of future progression to advanced EAC versus the risk for death due to other causes.

What is an acceptable surveillance interval after ER monotherapy? We detected all progressors at early stages, and we found no progression within the first 18 months post-ER. This suggests that annual surveillance is an accepted strategy and that we can safely perform the first FU endoscopy 1 year after confirmation of a completely flat BE post-ER. On the other hand, in older patients with a short remaining BE segment, we may stop endoscopic FU directly after ER, based on the aforementioned considerations.

Finally, if a patient has predictors for a low success chance after RFA, such as BE regeneration of the ER scar or a long BE without any squamous islands 26, one may decide not to start RFA but perform surveillance instead, independent of a patient’s life expectancy.

This is the first study that provides long-term FU data for an alternative treatment strategy in older patients with BE-related neoplasia. In our cohort of patients treated in a centralized setting by experienced endoscopists, this constituted 10% of the population that qualified for RFA after ER according to current guidelines. The suggested ER monotherapy strategy is advised in patients with a life expectancy of < 5–10 years and should be considered for a life expectancy of < 15–20 years. We suggest to consider and discuss this strategy in patients aged > 70 years and those with severe comorbidity.

Some limitations need to be addressed. The median duration of endoscopic FU was 21 months, while the median time to progression was 26 months, and all progressors occurred at minimal 18 months after ER. In light of this, we performed analysis that only included patients with FU over 18 months, which showed a minimally increased annual progression risk (i.e., 8.0% for all patients and 8.6% for patients with FU > 18 months). Still, if we would have had longer endoscopic FU, the annual progression risk might potentially have increased with a peak after longer FU, suggesting that the progression risk is not constant over time but increased over the years. Unfortunately, our data are too limited for solid analysis of this aspect. On the other hand, we report results for patients with *limited life expectancy*, and ultra-long-term FU data therefore have no clinical consequences. This is reflected by the fact that only one-third of patients was still under endoscopic surveillance at the moment of data collection. The others had already died of other causes (one-third) or were alive after endoscopic FU was already stopped (one-third). Therefore, extended FU with a potentially higher progression rate would not have changed the long-term outcomes, mortality rates, or our conclusions and recommendations.

Other limitations include the low number of events to assess predictive factors, which limited us to perform univariable analysis only. The FU duration for vital status may have been too short to detect recurrent, symptomatic disease among those patients whose FU was stopped early. A total of 7 patients had no endoscopic FU and were only assessed for vital status.

We are currently working on clinical prediction tools to provide individualized, evidence-based advices on optimal FU strategy after ER and/or RFA, taking account of the risk for progression and EAC-related death on the one hand, and patient age, comorbidity, and risk for unrelated death on the other. These data might help in defining the optimal strategy after ER monotherapy in the future.

In conclusion, ER monotherapy with endoscopic surveillance of the residual flat BE is a valid alternative to prophylactic ablation therapy of residual BE, in selected patients.
